# Implementation of Second-Tier Tests in Newborn Screening for Lysosomal Disorders in North Eastern Italy

**DOI:** 10.3390/ijns5020024

**Published:** 2019-06-21

**Authors:** Alberto B. Burlina, Giulia Polo, Laura Rubert, Daniela Gueraldi, Chiara Cazzorla, Giovanni Duro, Leonardo Salviati, Alessandro P. Burlina

**Affiliations:** 1 Division of Inherited Metabolic Diseases, Regional Center for Expanded Neonatal Screening, Department of Women and Children’s Health, University Hospital of Padova, Via Orus 2/B, 35129 Padova, Italy; 2 Institute of Biomedicine and Molecular Immunology (IBIM), National Research Council, Via Ugo La Malfa, 153-90146 Palermo, Italy; 3 Clinical Genetics Unit, Department of Women’s and Children’s Health, University of Padova, Via Giustiniani 3, 35128 Padova, Italy; 4 IRP Città della Speranza, Corso Stati Uniti 4, 35129 Padova, Italy; 5 Neurological Unit, St. Bassiano Hospital, Via dei Lotti 40, 36061 Bassano del Grappa, Italy

**Keywords:** lysosomal expanded newborn screening, Pompe disease, Fabry disease, Gaucher disease, MPS I, biomarkers, second-tier test, GAGs, lysoGb1, lysoGb3

## Abstract

The increasing availability of treatments and the importance of early intervention have stimulated interest in newborn screening for lysosomal storage diseases. Since 2015, 112,446 newborns in North Eastern Italy have been screened for four lysosomal disorders—mucopolysaccharidosis type I and Pompe, Fabry and Gaucher diseases—using a multiplexed tandem mass spectrometry (MS/MS) assay system. We recalled 138 neonates (0.12%) for collection of a second dried blood spot. Low activity was confirmed in 62 (0.06%), who underwent confirmatory testing. Twenty-five neonates (0.02%) were true positive: eight with Pompe disease; seven with Gaucher disease; eight with Fabry disease; and two with Mucopolysaccharidosis type I. The combined incidence of the four disorders was 1 in 4497 births. Except for Pompe disease, a second-tier test was implemented. We conclude that newborn screening for multiple lysosomal storage diseases combined with a second-tier test can largely eliminate false-positives and achieve rapid diagnosis.

## 1. Introduction

Lysosomal storage diseases (LSDs) are inborn errors of metabolism related to lysosome impairment. LSDs, caused by the deficient or absent activity of a specific lysosomal enzyme or transporter, result in the accumulation of non-metabolised macromolecular substrates within lysosomes. When LSDs are suspected, biochemical assays demonstrating the accumulation substrate such as lysosphingolipids (LysoGb1 for Gaucher disease, LysoGb3 for Fabry disease, LysoSM and LysoSM509 for Niemann-Pick diseases A/B and C), glycosaminoglycans (GAGs) for MPSs and oligosaccharides for Pompe (tetrasaccharide), enzyme analysis in leukocytes/lymphocytes and gene analysis allow to confirm the diagnosis [1,2].The increasing availability of therapies and the importance of early intervention have stimulated interest in the use of newborn screening (NBS) to diagnose these disorders.

Since Chamoles and co-workers showed that most lysosomal enzymes are active in DBS, thus permitting their activities to be measured with fluorimetric substrates [3], the development of assays for testing LSDs using DBS reached considerable progress in the last decade. Nowadays, the development of fluorimetry coupled with “digital microfluidics” (DMF) and electrospray ionization tandem mass spectrometry (MS/MS) for detecting LSDs allow a simultaneous quantification of several enzymes’ activity [4,5], thus permitting the neonatal diagnosis of Gaucher, Pompe, Fabry, MPS I, Niemann-Pick type A/B and Krabbe diseases.

In Italy, a pilot NBS for LSDs began in 2005, when approximately 37,000 Italian newborns were screened for Fabry disease with a fluorometric assay [6]. In the same period, screening for Pompe disease was begun in Taiwan, followed a few years later by screening for Fabry disease [7,8]. In the United States, in 2005, New York was the first state to screen newborns for an LSD (Krabbe disease) via multiplexed tandem mass spectrometry (MS/MS) [4,9]. Subsequently, in Austria, a seminal NBS pilot study showed the feasibility of NBS for several LSDs using enzymatic activity analysis in dried blood spots (DBSs) [10]. In 2013, a pilot program for multiple LSDs was carried out in Missouri, encompassing Pompe disease, Gaucher disease, Fabry disease and mucopolysaccharidosis type I (MPS I) [11]; LSD frequency was found to be 1 in 2081. In the last five years, several pilot LSD screening programs have been implemented in a number of countries worldwide, showing that their extension to large populations is feasible and economically justifiable [12,13,14]. Notably, the largest program for LSD screening in DBSs, performed in Illinois (USA), determined a total incidence of 1 in 7849 neonates screened [12]. Nevertheless, the choice of disease panels for expanded NBS programs is still controversial, as reflected by the diverse disease panels used in screening programs around the world. However, the Recommended Uniform Screening Panel (RUSP) of the US Department of Health and Human Services Secretary’s Advisory Committee on Heritable Disorders in Newborns and Children recently included Pompe disease and MPS I in the primary panel for neonatal screening programs [15].

In 2015, we implemented the neonatal screening programme mandated by Regional Laws including MPS I, Pompe, Fabry and Gaucher diseases [16]. The unexpected high number of screen-positive specimens reported in all the previous studies highlights the need to introduce a second-tier testing in order to reduce the recall rate. Enzyme measurement in leukocytes/lymphocytes and mutation analysis does not always allow to distinguish affected patients from pseudo deficient variants. Recently, lysosphingolipids in DBS have been proposed as diagnostic biomarkers for Gaucher disease (LysoGb1) [17], Fabry disease (LysoGb3) [18] and in MPS I the GAGs concentrations [19].

The LSDs panel screening is included in the expanded neonatal screening programme mandated by Regional Laws [20,21]. Here, we report our three-year experience of over 110,000 neonates screened for LSDs and specifically discuss the role of biomarkers as second-tier tests in a newborn LSD screening program.

## 2. Materials and Methods

### 2.1. Study Population

Newborns’ DBS samples were consecutively collected from September 2015 to February 2019 at the Regional Center for Expanded Newborn Screening, University Hospital of Padua. Informed consent was obtained from a parent. The Center is running an expanded NBS program for infants born in the North East of the Italy, comprising approximately 35,000 newborns per year. The DBSs were assayed for Fabry disease (deficiency in α-galactosidase (GLA)), Pompe disease (deficiency in α-glucosidase (GAA)), Gaucher disease (deficiency in β-glucocerebrosidase (ABG)) and MPS I (deficiency in α-l-iduronidase (IDUA)).

According to the NBS protocol, samples were collected at 48 h of life on the same card used for the other NBS tests; a second sample was required for premature infants (<34 gestational weeks and/or weight <2000 g) and for ill newborns (i.e., those receiving transfusions or parenteral nutrition).

### 2.2. LSD Screening Assay

GLA, GAA, IDUA and ABG enzyme activities were determined in a single DBS by stable isotope dilution flow injection analysis MS/MS (FIA-MS/MS) using anNeoLSD kit (PerkinElmer, Turku, Finland). Flow injection analyses were performed using an Acquity TQD and a Xevo TQ MS (Waters Corp., Milford, MA, USA). Enzyme activities are expressed as μmol/L/h (μM/h). As previously described, the recall cutoff was established at a Multiple of the Median (MoM) of 0.2 [12].

### 2.3. Second Tier Tests

DBS Lysosphingolipids (LysoGb1 or LysoGb3) were measured by LC-MS/MS as we recently described [17]. Lysosphingolipids were extracted from a 3.2 mm diameter DBS with 100 μL of a mixture of ethanol:acetonitrile:water (80:15:5, *v*/*v*) containing internal stable isotope standards (LysoGb1–d5). Chromatographic separation was performed using a C18 column with a gradient of water and acetonitrile both with 0.1% formic acid in a total run time of 4 min. The compounds were detected in the positive ion mode electrospray ionization (ESI)-MS/MS by multiple reaction monitoring (LysoGb3 786.5>282.3; LysoGb1 462.3>282.3; LysoGb1–d5467.3>287.3). DBS GAGs levels were measured by LC-MS/MS after methanolysis according to Zhang et al. with minor modifications [22]. Two DBS punching were incubated with methanolic-HCl 3 N solution at 65 °C for 45 min in an orbital shaker. Chromatographic separation was performed using an Amide column with a gradient of ACN and water with 10 mM Ammonium Acetate in a total run time of 9 min. Method was validated analyzing six paediatric patients with confirmed MPS I. The spots were collected after diagnosis and all samples had high levels of GAGs.

### 2.4. Confirmatory Testing

Newborns with a positive LSD screening result were referred to the Division of Inherited Metabolic Diseases for confirmatory testing that included clinical evaluation, mutational analysis, substrate quantification and/or enzyme activities in leucocytes/lymphocytes. For Pompe disease, the assessments included electrocardiography, echocardiography, urinary tetrasaccharide (Glc4) on LC-MS/MS [23] and blood tests, including creatine phosphokinase (CPK), aspartate aminotransferase (AST) and alanine aminotransferase (ALT). Plasma glucosylsphingosine (LysoGb1) was assayed for Gaucher disease [24], whereas plasma globotriaosylsphingosine (LysoGb3) [24] and urinary GAGs were analysed for Fabry disease and MPS I, respectively [25].

## 3. Results

Between September 2015 and February 2019, 112,446 newborns were screened for the four LSDs. 138 neonates (0.12%) had an enzyme activity below 0.2 MOM and were recalled for collection of a second DBS (Table 1). Low activity was confirmed in 62 (44.9%) neonates, who subsequently had confirmatory testing that includes clinical studies and diagnostic biochemical and mutation analyses. The positive screening tests, recall rates and patients with a confirmed diagnosis are summarised in Table 1.

In the Pompe disease screening (Table 2), 18 neonates were identified as high risk for the disorder. After confirmatory testing, eight of them were confirmed to be affected. Two newborns, presenting the lowest GAA activity at the NBS (0.2 and 0.45 µM/h, 1.4% and 3.1% of the normal mean, respectively) showed hypertrophic cardiomyopathy at birth. Subsequent analysis of urinary Glc4 was positive. The six patients with a later-onset form of Pompe disease (late-onset Pompe disease) had a GAA activity ranging from 4.2% to 13.4%. Mutational analysis revealed a high prevalence of the intronic mutation IVS1-13T>G. Patients homozygous for this mutation had normal biochemical tests for serum CPK, ALT, AST and urinary Glc4 excretion. In two patients who were compound heterozygous for IVS1-13T>G and the frameshift mutation p.P79RfsX12 that is associated with a very severe phenotype [26,27], CPK was elevated and Glc4 showed increased values without cardiac involvement. False-positive newborns were carriers for a single allelic pathogenic mutation, pseudodeficiency alleles or a variant of unknown significance. In this group, enzyme activity ranged from 9.8% to 18.6%.

In the MPSI screening, 52 were recalled for a second DBS. 26/52 with confirmed reduced IDUA enzyme activity underwent urinary GAG and mutation analysis. 2/26showedpathogenic mutations as reported in Table 3. MPS I was confirmed in two newborns with very low enzyme activity in the NBS (1.6% and 2.1% of the normal mean): both had high excretion of heparan sulphate (HS) and dermatan sulphate (DS) in urine. Upon mutational analysis, one was found to be compound heterozygous for two severe mutations (c.46-57del12/p.Y201X) and had clinical features of a severe Hurler phenotype; the second was homozygous for the mutation p.P533R, reported to be common in North African MPS I patients (Hurler to Hurler/Scheie phenotypes) [28,29]. The other 24 newborns recalled for low IDUA enzyme activity were subsequently negative on confirmatory testing, presenting normal urinary GAGs and/or variants known to be pseudodeficiency alleles (p.A361T, p.R263W, pD223N, p.A79T and p.S586F). A high prevalence of these alleles was found in newborns of African descent (18 of 24), with p.A79T the most commonone. The residual enzyme activity in these newborns overlaps that of true-positive MPS I, ranging from 1% to 12.1% (mean, 7.4%) of the normal mean. This finding makes it difficult to classify the risk based only on this parameter.

**Figure 1:**
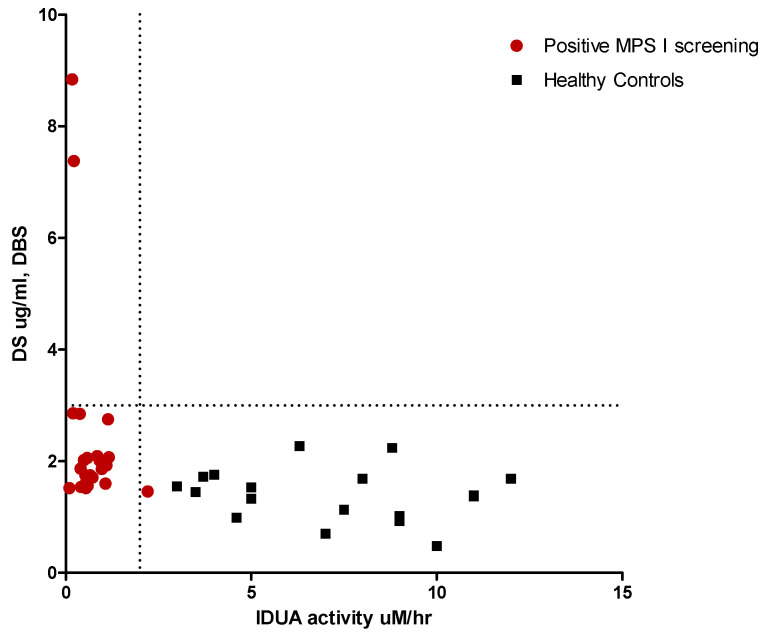
DBS-GAGs as second tier test for IDUA screening. Dermatan sulphate in DBS vs IDUA activity in newborns with suspicion of MPS I and healthy neonates.

To improve the performance of NBS for MPS I and reduce the large number of recalls, we recently developed a second-tier test based on quantification of GAG levels in DBSs. HS and DS were assayed by methanolysis followed by LC-MS/MS with a method optimized for DBSs based on that developed by Zhang et al. [22]. Newborn DBS screening of infants with pseudodeficiency and true MPS I was retrospectively analyzed to determine GAG levels (Figure 1). Heparan and dermatan sulphate were elevated only in the two samples carrying the pathogenic mutations (HS 4.9–10.4 µg/mL respectively, normal values 0–3.2; DS 7.4–8.8 µg/mL respectively, normal values 0.5–2.7), and normal profiles in all samples showed a pseudodeficiency activity. Therefore, now we used the DBS GAGs methodology instead of urinary GAGs analysis.

In the Gaucher disease screening, 28 newborns were recalled for a second DBS. 7/28 were identified as high risk for the disorder because reduced ABG activity (<2 µM/h) and accumulation of LysoGb1 (>31.1 nmol/L, [17]) and successively confirmed by mutation analysis (Table 4). p.N409S and p.L483P were the most common mutations in our cohort, as in the Gaucher disease Italian population [30]. In this study, we identified two neonates with compound heterozygous p.N409S/p.L483P, one with p.N409S/p.N227S and three who were homozygous for p.N409S. In one newborn, only one mutation was found (p.N409S/not found); however, high LysoGb1 plasma levels (53.5 nmol/L, normal values 1.15–3.25 nmol/L) indicated Gaucher type I.

In the Fabry disease screening, 23 newborns were recalled for a second DBS. 11/23hadconfirmed reduced GLA enzyme activity at the recall DBS. 8/11 newborns showed pathogenic mutations associated with type 2 Fabry disease (late-onset) and GLA activity ranging from 6.1% to 14.4% of the mean activity for normal population [31]. At the same time of the mutation analysis study, we tested LysoGb3 in plasma and in 5/8 the levels were slightly elevated (mean 1.12 nmol/L, range 0.54–2.17 nmol/L; normal value <0.46 nmol/L). 3/11 newborns were found to be hemizygous for unreported variants of unknown significance or benign mutations (i.e., p.A143T) and had a GLA activity ranging from 8.3% to 19.6% of that of the mean for normal population and normal levels of plasma LysoGb3 (Table 5).

## 4. Discussion

The LSD NBS program in North East Italy was initiated on 1 September, 2015. Since then, 112,446 newborns have been screened for Pompe disease, MPS I, Gaucher disease and Fabry disease.

As previously reported [12,13], our experience confirms that screening for LSDs is feasible and appears to effectively detect positive cases. The multiplexed NeoLSD^®^ assay system (PerkinElmer) for Pompe, Fabry, Gaucher and MPS I was the most adaptable and flexible testing system for the expansion of NBS programs. The use of Flow Injection Analysis coupled with MS/MS (FIA-MA/MS) does not constitute a large increase in instrument complexity. Because the method needs an overnight incubation, the data are available in the morning of day 2. With this in mind, each laboratory has to evaluate the requirements for same-day data regarding Pompe disease. This reflects the experience of other investigators who have used a similar MS/MS methodology to identify newborns at risk of LSDs [1].

The combined frequencies of the four LSDs identified in our study are similar to those detected in our previous report [16], the total incidence decreasing slightly from 1 in 4411 to 1 in 4497. The most relevant changes concerned Fabry disease type 2(late-onset), from 1 in 8882 to 1 in 14,056, Pompe disease (1 in 22,205 to 1 in 14,056) and Gaucher disease (from 1 in 22,205 to 1 in 16,063). For MPSI, the incidence has been approximately confirmed: from 1 in 44,411 to 1 in 56,223, making it the rarest of the four diseases.

Our data confirmed the high incidence of LSDs in the Italian population, as reported in a retrospective survey [32]. In the past, with some exceptions due to specific geographical clusters, the genetic background of the Italian population has been relatively homogenous and indicative of a population with a low consanguinity rate. The above mentioned study was carried out almost than 20 years ago, no particular geographic area or ethnic group was at a higher risk for LSDs. Nowadays, our data show that the recent immigration from Africa and Asia reflected a different population profile in comparison with that we previously reported [32]. Many of our LSD patients from North-Africa, showed high consanguinity [33].

Our results showed a high incidence of false-positive patients in LSD newborn screening. Due to that, we took the decision to evaluate the use of biomarkers quantification as second-tier analysis for MPS I, Gaucher and Fabry diseases. This allows for better differentiation between patients with pathogenic mutations, pseudodeficiency alleles, and/or benign variants at the time of screening, which may reduce the number of false positives.

Plasma LysoGb1 has been proposed as a high selective biomarker for clinical use in Gaucher disease. LysoGb1 measured by LC-MS/MS in DBS has recently proposed as a very useful second-tier test biomarker [17,34]. Furthermore, in Gaucher disease, LysoGb1 has been shown to increase over time, but it has been proposed as a useful biomarker also in prenatal diagnosis [24,35]. Our experience shows that LysoGb1 is elevated since birth. Moreover, all the neonates with increase LysoGb1 were confirmed as true positive for Gaucher disease, with a positive predictive value of 100%. According to these findings, we propose to use LysoGb1 as possible second-tier biomarker.

Plasma LysoGb3 has a high diagnostic sensitivity, especially for classical male Fabry patients [36]. The use of this marker in neonatal period is very limited. There are three reports in which LysoGb3 was evaluated in the neonatal period: Johnson et al. reported a pioneering work on LysoGb3 in DBS collected from Fabry disease neonates detected by newborn screening [18]. Concentrations of LysoGb3 were below the limit of quantitation in most newborn infants with Fabry Disease. Spada et al. reported one single male patient with classical form of Fabry with highly elevated levels since birth [37]. Chien et al. confirmed that increased LysoGb3 values in neonatal period are very suggestive of Fabry disease, but normal LysoGb3 levels cannot exclude the possibility of Fabry disease [38]. In our study, the concentrations of LysoGb3 in DBS were normal in most newborns (except two). All our patients carried mutations associated with Type 2 Fabry disease in which LysoGb3 has been already reported in the normal range [37].

As we recently reported [17], concentrations in both the plasma and DBS of LysoGb1 and LysoGb3 were highly correlated and able to identify the affected patients.

The use of glycosaminoglycans (GAGs) as biomarkers for the diagnosis of neonatal MPS has been recently proposed by Kubaski as a first tier test [39]. Retrospective analysis of GAGs DBS levels in our study showed elevated levels of heparan and dermatan sulphates in the two patients carry pathogenic mutations and normal in all patients with pseudodeficiency alleles. Since urinary GAGs can be elevated due to other conditions unrelated to MPS (i.e., kidney immaturity), we proposed the GAGs dosage in DBS as second-tier test in patients with low enzyme activity.

LSD diseases with both clinical onsets—early and late—can be diagnosed with NBS. Early-onset presentation showed an incidence of 1 in 53,777 newborns for Pompe and MPS I, respectively. These forms are those that most benefit from early diagnosis and early treatment. In the two newborns with severe infantile-onset Pompe disease, we were able to start enzyme replacement therapy within two weeks of life (5 and 12 days, respectively). The two newborns with MPS I were also treated with enzyme replacement therapy. The newborn with the Hurler form received a bone marrow transplantation at six months of age.

The late-onset forms globally occur in one newborn every 5975. The diagnosis of newborns with the late-onset phenotypes represents an important issue for both physicians and parents. Decades can pass before the disease shows signs and symptoms, and patients and family members can experience anxiety after the screening result. On the other hand, patients identified in our program with late-onset disorders will receive clinical follow-up and, in some cases, early diagnosis through NBS may eliminate the ‘diagnostic odyssey’ experienced by many patients in the past. Moreover, the clinical follow-up will allow timely treatment as soon as clinical manifestations appear.

We regularly follow-up patients with Gaucher and Fabry disease that typically manifest symptoms later in the life. Till now, none of the patients showed clinical manifestations and needed therapeutical intervention.

Any newborn screening may raise ethical issues. This is true also for lysosomal diseases, either because of early therapy application either because of delayed clinical manifestations for later-onset clinical types (i.e., Fabry disease). In our setting dedicated to inherited metabolic diseases, a team which includes pediatric clinicians, a geneticist, an adult neurologist, and a psychologist are working towards the most informed communication. This approach is useful, especially in the case of Fabry disease whereas the diagnosis of benign variants and variants of unknown significance can be particularly difficult to understand, as already reported in the literature [40]. Difficulties are also encountered with young asymptomatic mothers who feel uncomfortable to be tested and periodically monitored.

We conclude that neonatal screening for multiple LSDs is effective in identifying neonates at risk for LSD in a large population scale. Our data proposed the use of second-tier test in DBS with reduced enzyme activities for Gaucher disease and MPS I, while in Fabry disease further studies are needed. Optimised cut-off values combined with a second-tier test, where available, could largely reduce false-positive rate and therefore decrease the follow-up burden on clinics and families.

## Figures and Tables

**Table 1:** Newborn screening results, recalls and confirmed diagnosis.

LSD	Total Screened	Positive NBS	Recall Rate	Patient Undergo to Confirmatory Testing	Patients with Confirmed Disorder	Pseudo Deficit	VUS	Carrier Status
Gaucher	112,446	28	0.03	7	7	0	0	0
Pompe	112,446	28	0.03	18	8	7	2	1
Fabry	112,446	23	0.02	11	8	1	2	0
MPS I	112,446	52	0.05	26	2	20	2	2
Multiple LSD	112,446	7	0.01	0				
Total	112,446	138	0.12	62	25	28	6	3

**Table 2:** NBS enzyme activity, confirmatory testing results and final diagnosis in newborns with suspicion of Pompe Disease. Newborns were ordered based on neonatal screening enzyme activity.

Pompe Disease	NBS Enzyme Activity	% Enzyme Activity	Sex	Ethnic Origin	Biochemical Test *	Glc4 ** (mmol/mol creat)	Genotype	Predicted Phenotype
GAA-01	0.2	1.4	M	West Africa	CPK 1063 ALT 15 AST 112	71.2	p.R854X/NF	IOPD
GAA-02	0.45	3.1	F	European	CPK 990 ALT 69 AST 128	94.4	p.D645N/p.W746X	IOPD
GAA-03	0.61	4.2	M	North Africa	CPK 448 ALT 48 AST 98	NP	IVS1-13T>G/p.P79RfsX12	LOPD
GAA-04	1.03	7.1	M	European	Normal	0.5	IVS1-13T>G/IVS1-13T>G	LOPD
GAA-05	1.11	7.6	M	European	Normal	NP	IVS1-13T>G/IVS1-13T>G	LOPD
GAA-06	1.17	8.0	M	European	Normal	0.2	IVS1-13T>G/IVS1-13T>G	LOPD
GAA-07	1.41	9.7	F	North Africa	CPK 682 ALT 68 AST 130	20.1	IVS1-13T>G/p.P79RfsX12	LOPD
GAA-08	1.43	9.8	M	European	Normal	NP	p.V222M/p.V222M	Pseudo
GAA-09	1.84	12.6	M	European	NP	NP	p.R385H_p.E689K_p.A704T_p.W746C	Pseudo
GAA-10	1.89	13.0	M	Asia	Normal	NP	p.D645E/wt	Carrier PD
GAA-11	1.92	13.2	M	Asia	Normal	NP	p.G576S/p.E689K	Pseudo
GAA-12	1.94	13.3	M	European	Normal	6.3	IVS1-13T>G/IVS1-13T>G	LOPD
GAA-13	1.97	13.5	M	European	Normal	NP	IVS1-13T>G /p.G576S/p.E689K	Pseudo
GAA-14	2.11	14.5	M	Asia	Normal	12.7	p.W746C/wt	Carrier PD
GAA-15	2.22	15.3	F	European	Normal	NP	p.R178H/p.R178H	Pseudo
GAA-16	2.27	15.6	F	South Asia	Normal	NP	IVS1-13T>G /p.G576S	Pseudo
GAA-17	2.52	17.3	M	European	Normal	0	p.V222M/p.V222M	Pseudo
GAA-18	2.7	18.6	M	European	Normal	NP	IVS1-13T>G /p.G576S/p.E689K	Pseudo

* Normal values: CPK 0-295 U/L, ALT 5-40 U/L, AST 9-80 U/L. ** Glc4 <16.3 mmol/mol creat. IOPD, Infantile Onset Pompe Disease; LOPD, Late Onset Pompe Disease. NF, Not Found; NP, Not Performed; Pseudo, Pseudodeficiency; VUS, Variant of Unknown Significance.

**Table 3:** NBS enzyme activity, confirmatory testing results and final diagnosis in newborns with suspicion of MPS I. Newborns were ordered based on neonatal screening enzyme activity.

MPS I	NBS Enzyme Activity	% Enzyme Activity	Sex	Ethnic Origin	Urinary GAGs (mg/mmol creat)	Genotype	Predicted Phenotype
IDUA-01	0.1	0.9	M	West Africa	NP	p.A79T/p.A79T	Pseudo
IDUA-02	0.17	1.6	F	European	POS (DS 90.6 HS 220.6)	p.S16_A19del/p.Y201X	MPS I H
IDUA-03	0.2	1.9	M	South Asia	NORMAL	NP	Pseudo
IDUA-04	0.22	2.1	F	North Africa	POS (DS 121.7 HS 215.3)	p.P533R/p.P533R	MPS I H H/S
IDUA-05	0.38	3.6	F	North Africa	NORMAL	p.R628G/p.R628G	VUS
IDUA-06	0.4	3.8	M	West Africa	NORMAL	p.A79T/p.D223N	Pseudo
IDUA-07	0.41	3.9	M	North Africa	NORMAL	p.A79T_ p.A361T/p.Y581X	Carrier/Pseudo
IDUA-08	0.49	4.6	F	West Africa	NORMAL	p.A79T/p.A79T	Pseudo
IDUA-09	0.53	5.0	F	West Africa	NP	p.A79T/p.D223N	Pseudo
IDUA-10	0.54	5.1	M	West Africa	NP	p.A79T_p.T99I/p.D223N	Pseudo
IDUA-11	0.55	5.2	F	European	NORMAL	p.L526P/p.L526P	VUS
IDUA-12	0.58	5.5	F	West Africa	NP	p.A79T/p.A361T	Pseudo
IDUA-13	0.59	5.6	F	North Africa	NORMAL	p.R263W/p.P650L	Pseudo
IDUA-14	0.66	6.3	F	West Africa	NORMAL	p.A79T/p.A79T	Pseudo
IDUA-15	0.71	6.8	F	European	NP	p.S16_A19del/p.H82Q	Carrier/Pseudo
IDUA-16	0.72	6.9	M	West Africa	NORMAL	p.A79T/p.A79T	Pseudo
IDUA-17	0.85	8.1	M	North Africa	NORMAL	p.A79T/p.R263W	Pseudo
IDUA-18	0.88	8.4	M	NA	NORMAL	NP	Pseudo
IDUA-19	0.92	8.8	F	West Africa	NORMAL	p.A79T/p.V322E	Pseudo
IDUA-20	0.97	9.3	M	West Africa	NORMAL	p.A79T/p.F501L	Pseudo
IDUA-21	1.07	10.2	M	NA	NORMAL	p.A79T/p.R263W	Pseudo
IDUA-22	1.1	10.5	M	West Africa	NORMAL	p.A79T/p.S586F	Pseudo
IDUA-23	1.14	10.9	F	West Africa	NORMAL	p.A79T/p.A79T	Pseudo
IDUA-24	1.16	11.1	M	North Africa	NP	p.R263W/p.S586F	Pseudo
IDUA-25	1.26	12.0	M	European	NORMAL	NP	Pseudo
IDUA-26 *	2.21	21.1	F	West Africa	NP	p.A79T/wt	Pseudo

MPS I H, MPS I Hurler; MPS I H/S, MPS I Hurler-Scheie; HS, Heparan Sulphate; IDUA, acid α-l-iduronidase; DS, Dermatan Sulfate; NA, Not Available; NP, Not Performed; Pseudo, Pseudodeficiency; VUS, Variant of Unknown Significance. Urinary GAGs normal values: DS <38.1 mg/mmol creat, HS <4.6 mg/mmol creat. * IDUA 26 was tested because she was twin of IDUA 09.

**Table 4:** NBS enzyme activity, confirmatory testing results and final diagnosis in newborns with suspicion of Gaucher Disease. Newborns were ordered based on neonatal screening enzyme activity.

Gaucher Disease	NBS Enzyme Activity	% Enzyme Activity	Sex	Ethnic Origin	Second Tier DBS LysoGb1 ** (nmol/L)	Genotype	Predicted Phenotype
ABG-01	0.44	4.5	M	European	163.8	p.N409S/p.L483P	GD I
ABG-02	0.6	6.2	M	European	135.9	p.N409S/p.N409S	GD I
ABG-03	0.88	9.0	F	European	77.4	p.N409S/p.L483P	GD I
ABG-04	1.07	11.0	M	European	114.8	p.N409S/p.N409S	GD I
ABG-05	1.07	11.0	F	European	116.6	p.N409S/p.N227S	GD I
ABG-06	1.26	12.9	M	European	186.0	p.N409S/Not found	Likely GD I
ABG-07	2.01	20.6	M	European	63.1	p.N409S/p.N409S	GD I

ABG, acid β-glucocerebrosidase; GD I, Gaucher Disease Type I. ** DBS LysoGb1 normal values: 2.3–31.1 nmol/L [17].

**Table 5:** NBS enzyme activity, confirmatory testing results and final diagnosis in newborns with suspicion of Fabry Disease. Newborns were ordered based on neonatal screening enzyme activity.

Fabry Disease	NBS Enzyme Activity	% Enzyme Activity	Sex	Ethnic Origin	Second-Tier DBS LysoGb3 * (nmol/L)	Genotype	Predicted Phenotype [31]
GLA-01	0.64	6.1	M	European	1.02	p.N215S	Type 2 FD
GLA-02	0.72	6.9	M	North Africa	1.79	p.R363H	Type 2 FD
GLA-03	0.73	7.0	M	European	2.98	p.R356G	Type 2 FD Likely
GLA-04	0.77	7.3	M	East Asia	0.75	IVS4+919G>A	Type 2 FD
GLA-05	0.79	7.5	M	European	0.41	p.M290L	Type 2 FD
GLA-06	0.87	8.3	M	European	0.73	p.G116A	VUS (Not reported)
GLA-07	1.16	11.1	M	East Asia	0.62	IVS4+919G>A	Type 2 FD
GLA-08	1.28	12.2	M	European	1.06	p.L286V	VUS (Not reported)
GLA-09	1.37	13.1	M	European	0.83	p.M51I	Type 2 FD
GLA-10	1.51	14.4	M	West Africa	0.96	p.R356Q	Type 2 FD
GLA-11	2.05	19.6	M	European	0.54	p.A143T	Benign

GLA, acid α-galactosidase; * DBS LysoGb3 normal values: <1.45 nmol/L [17].
